# Feco-prevalence and risk factors of *Helicobacter pylori* infection among symptomatic patients at Dessie Referral Hospital, Ethiopia

**DOI:** 10.1186/s12879-018-3179-5

**Published:** 2018-06-07

**Authors:** Abdurahaman Seid, Wondmagegn Demsiss

**Affiliations:** Department of Medical Laboratory Science, College of Medicine and Health Sciences, Wollo University, P.O. Box 1145, Dessie, Ethiopia

**Keywords:** *H. pylori*, Stool antigen, Serum IgG, Risk factors, Ethiopia

## Abstract

**Background:**

Helicobacter pylori (*H. pylori*) infection is the most common chronic bacterial infection in the world. It can result in various upper gastroduodenal diseases. The prevalence varies among countries, population groups within the same country and testing methods. The aim of the study was to determine feco-prevalence and risk factors of *H.pylori* infection among symptomatic patients in Amhara region, Northeast Ethiopia.

**Methods:**

A cross sectional study was conducted in a total of 342 new consecutive outpatients with upper abdominal complaints at Dessie Referral Hospital from May to July, 2016. A structured questionnaire was used to collect the socio-demographic, lifestyle and environmental data. Stool samples were used to detect *H. pylori* specific antigen. Blood samples were assessed for anti-*H. pylori* IgG and ABO blood types. SPSS version 20.0 statistical software package was used for data analysis. Chi-square test and logistic regression were used in the analysis and *P*-value ≤0.05 was considered as statistically significant.

**Results:**

*H. pylori* stool antigen and serum anti-*H.pylori* IgG detection rate was 30.4 and 60.5% respectively with kappa measure of agreement of 0.271. Antigen detection was significantly associated with family size (> 3) [AOR = 1.83, 95% CI: 1.10–3.05, *p* = 0.02], more persons (> 3) sharing the same bed room in the household [AOR = 2.91, 95% CI: 1.39–6.11, *p* = 0.005], alcohol consumption (> once a week) [AOR = 2.70, 95% CI: 1.49–4.89, *p* = 0.001] and individuals’ blood type: group O [AOR = 8.93, 95%CI: 1.79–44.48, *p* = 0.008] and group A [AOR = 5.53, 95%CI: 1.08–28.36, *p* = 0.040]. Gender, age, marital status, occupation, educational level, residence, smoking as well as coffee, tea, fruits and vegetables consumption were not statistically associated with *H. pylori* antigen detection (*p* > 0.05).

**Conclusion:**

The overall *H. pylori* stool antigen and anti-*H. pylori* IgG detection rate was 30.4 and 60.5%, respectively. The test agreement was not strongly convincing and needs further evaluation. Alcohol consumption, overcrowding and ABO blood group were significantly associated with *H. pylori* antigen detection.

## Background

*H. pylori* infects about half of the worlds’ population with higher prevalence in developing than developed countries [1–3]. The prevalence of *H.pylori* infection varies among societies and geographical locations, and is influenced by different factors like environmental factors, socio-demographic characteristics, socioeconomic status, hygienic practice and life style of the population [1, 2, 4]. *H. pylori* infection occurs with an exposure to the bacterium through oral–oral and fecal–oral route [4, 5], but the fecal-oral route was identified to be the most important route of transmission [4].

Besides large numbers of infected individuals remained as asymptomatic [6], *H. pylori* infection is a major public-health problem both in developed and developing nations. It is the leading cause of different types of upper gastroduodenal tract diseases such as gastric and duodenal ulcer, gastritis and Mucosal-Associated Lymphoid Tissue (MALT) lymphoma [3, 7]. It is also responsible for the development of gastric cancer [8] which is the second leading cause of cancer-related deaths worldwide [9].

Early detection of *H. pylori* infection using a reliable diagnostic method is important for the successful management and prevention of disease complications. Since the discovery of *H. pylori*, both invasive and noninvasive tests are used for the diagnosis of infection. Histology, culture, polymerase chain reaction (PCR) and rapid urease test (RUT) are invasive tests that ‘require biopsy specimens [10], whereas stool antigen test, Urea Breath Test (UBT) and serology are the most commonly used noninvasive tests [11, 12].

Even though a variety of diagnostic methods are available, only tests with sensitivity and specificity exceeding 90% are recommended for clinical practice [13]. Even though serology is most commonly used in developing countries it is not appropriate for the diagnosis of active *H. pylori* infection. This is due to its low specificity (79–83%), and presence of high titer of IgG for years after eradication of infection. This limits its importance to differentiate past and current exposure to *H. pylori* [2, 14]. However, the UBT and *H. pylori* stool antigen (HpSA) test are a reliable noninvasive method for detection of active and recent *H. pylori* infection [15, 16]. In the absence of UBT, the HpSA test is usually recommended for diagnosis of *H. pylori* infection [17] with a globally observed sensitivity and specificity of 94 and 97%, respectively [18].

Most previous Ethiopian studies employed serology for diagnosis of *H. pylori* infection and reported higher seroprevalence of *H. pylori* infection. It is documented that besides lack of specificity, serological test cannot differentiate past and current infection. This puts a major limitation to know the actual incidence and real association of *H. pylori* infection with potential risk factors. Some studies conducted in the Southern and Central part of Ethiopia reported the feco-prevalence and possible risk factors of *H. pylori* infection [19–22]. However, the reports lack consistency in prevalence and associated factors of *H.pylori* infection. This needs additional studies in order to document local burden of *H. pylori* infection and its associated risk factors.

Furthermore, feco-prevalence of *H. pylori* infection was not documented in the Amhara region of the country, which is quite different in socio-cultural properties of the population, environmental and geographical characteristics as compared to Southern and Central regions of the country. These factors may have an impact on the epidemiology of the disease. Currently, serology is the most commonly used rapid test algorithm for diagnosis of *H.pylori* infection in Ethiopia. It is valuable for each locality and region to determine the prevalence, identify risk factors associated with the infection and document the role of stool antigen test in comparison to commonly used rapid serologic test. Therefore, the aim of this study was to determine feco-prevalence and risk factors of *H. pylori* infection among patients with upper abdominal complaints. It also aimed to compare a rapid stool antigen and serologic test for the diagnosis of *H. pylori* infection.

## Methods

### Study area

The study was conducted at Dessie Referral Hospital, Dessie town, Amhara Region, North East Ethiopia. Dessie is located about 400 km from the capital of Ethiopia, Addis Ababa. Dessie Referral Hospital is the largest public hospital in the town. Patients with clinical indications of *H. pylori* infection are routinely tested in the hospital using rapid serological diagnostic tests.

### Study design and period

A cross-sectional study was conducted from May to July, 2016.

### Sample size and sampling technique

The sample size was determined by using single population proportion formula taking 72.2% prevalence of *H. pylori* infection among dyspeptic patients [23] with a marginal error of 5, and 95% confidence interval. Accordingly, the total sample size was determined to be 339 with 10% non-response rate. However, during the study period 342 new patients with clinical indications of *H. pylori* infection were examined at the outpatient department of the hospital and included consecutively. All patients who presented with recurrent upper gastro-intestinal tract symptoms of more than one-month duration were included in the study. Patients treated with any antibiotics, colloidal bismuth compounds, proton pump inhibitors (PPI) or H2 blockers within the last 4 weeks of study enrolment were excluded.

### Data collection and laboratory methods

#### Questionnaires

A structured and pre-tested “Amharic version” (local language) questionnaire was used for data collection. Prior to data collection the questionnaire was administered to 15 participants with upper gastrointestinal symptomatic patients to assess the clarity and understandability of questions. The questionnaire was administered for each adult participant/children guardian to collect information on the socio-demographic characteristic, hygienic practices and lifestyle factors such as alcohol, tea, coffee, raw fruits and vegetables consumption as well as cigarette smoking. For participants who can’t read and write, interview was employed.

### Clinical sample collection and analysis

#### ABO blood grouping and serological detection of *H. pylori*

About 5 ml of venous blood was collected from each participant aseptically through venipuncture and Anti- *H. pylori* antibody (IgG) detection as well as ABO blood grouping was made. Immediately after collection some portion of whole blood was used to determine ABO and Rh blood group by slide agglutination test using monoclonal anti-A, anti-B, anti-AB and anti-D (Rh) antibodies according to manufacturer’s recommendation. Some portion of collected blood was allowed to clot in a test tube and centrifuged at 3000 RPM for 10 min and serum was separated. The antibody was detected by one-step rapid test device (Zhejiang Orient Gene Biotech Co., LTD, china; http://www.orientgene.com/asp-en/home/index.aspx). The appearance of color band on both test and control line was interpreted as positive but color band only on the control line is interpreted as negative result. All tests were performed following the manufacturer’s instructions.

#### *H. pylori* antigen detection

Small amount of fresh stool sample was collected from each participant in sterile, wide mouth and screw caped containers. The presence of *H. pylori* antigen was determined using monoclonal anti-*H. pylori* antibody conjugated with colloid gold nitrocellulose membrane strip (Rapid stool antigen test) based on a lateral flow chromatographic immunoassay technique. Specimens were tested using stool antigen test strip (Guangzhou Wondfo Biotech CO., Ltd., China; https://www.bloomberg.com/research/stocks/private) with sensitivity and specificity of 99.1 and 99.2%, respectively. Test results were read and interpreted according to the manufacturer’s instructions.

### Data analysis

Data was coded, entered and analyzed using SPSS Version-20 (SPSS INC, Chicago, IL, USA). Results were summarized using descriptive statistics. Cohen’s Kappa statistics was performed to assess the agreement of rapid serological test (anti-*H. pylori* IgG) and rapid stool antigen detection. Chi-square test and binary logistic regression analysis were performed to check the presence of association between dependent variable (presence of *H. pylori* antigen) and independent variables. Furthermore, possible risk factors with a *P* value of < 0.2 in univeriate analysis were selected for further stepwise analysis with multiple logistic regression model. The OR and 95% confidence interval (CI) were used to estimate the risk factors independently associated of *H. pylori* infection. Finally, a *P* value of ≤0.05 was accepted as statistically significant.

## Results

### Socio-demographic, lifestyle and hygienic characteristic of study participants

Out of 342 participants, 189 (55.3%) were females. Majority of study participants 114 (33.3%) were in the age group 31–40 years. The mean (±SD) age of participants was 36.61 ± 13.2 years with range 4 to 77 years. Majority of participants 218 (63.7%) were married and urban dwellers account 210 (61.4%). One hundred sixty-seven (48.8%) participants were living in a family having four or more members (Table 1).

**Table 1:** Feco-prevalence of *H. pylori* infection against socio demographic, characteristics of symptomatic patients in Dessie Referral Hospital, Northeast Ethiopia, 2016 (*n* = 342)

Variables		freq (%)	HpSA (+)		
			No (%)	χ 2	p-value
Sex	Male	153 (44.7)	43 (28.1)	0.70	0.404
	Female	189 (55.3)	61 (32.3)		
Age in years	≤ 20	30 (8.7)	7 (23.3)	1.60	0.809
	21–30	94 (27.5)	26 (27.7)		
	31–40	114 (33.3)	38 (33.3)		
	41–50	53 (15.5)	17 (32.1)		
	> 50	51 (14.9)	16 (31.4)		
Marital status	Married	218 (63.7)	65 (29.8)	0.1	0.752
	Not married	124 (36.3)	39 (31.5)		
Residence	Rural	132 (38.6)	41(31.1)	0.04	0.836
	Urban	210 (61.4)	63 (30)		
Occupation	Government	111 (32.5)	32 (28.8)	1.40	0.924
	Student	45 (13.2)	15 (33.3)		
	Farmer	69 (20.2)	24 (34.8)		
	Merchant	51 (14.9)	15 (29.4)		
	Housewife	53 (15.5)	15 (28.3)		
	Others	13 (3.8)	3 (23.1)		
Education	No legal education	53 (15.5)	18 (34.0)	4.31	0.366
	Grade 1–4	28 (8.2)	10 (35.7)		
	Grade 5–8	58 (17.0)	15 (25.9)		
	Grade9–12	78 (22.8)	29 (37.2)		
	College/university	125 (36.5)	32 (25.6)		
Person sharing the same bed room in the household	1	77 (22.5)	18 (23.4)	10.14	0.006*
	2 to 3	181 (52.9)	49 (27.1)		
	> 3	84 (24.6)	37 (44.0)		
Family size	1–3	175 (51.2)	41 (23.4)	8.25	0.004*
	> 3	167 (48.8	63 (37.7)		

Key: * = Significant associations exist

An assessment on the food habits of the study participants revealed that 67.8 and 61.7% had a habit of tea and coffee consumption respectively; 53.5% had never drank alcohol in their life and 58.8% had a habit of eating raw fruits and/or vegetables at least once a week. About 94.2% (322) of participants had a habit of hand washing before eating and after visiting toilet; 282 (82.5%) used tap water for daily consumption. The frequency of blood group O was 137 (40.1%) and 326 (95.3%) were with rhesus positive (Rh + ve) blood type (Table 2).

**Table 2:** Association of HpSA detection with respect to genetic, hygiene and lifestyle factors of symptomatic patients in Dessie Referral Hospital, Northeast Ethiopia, 2016 (*n* = 342)

Variables		Freq (%)	HpSA(+)	χ 2	*p*-value
			=no (%)		
Alcohol drinking	Never	183 (53.5)	48 (26.2)	6.26	0.044*
	Once a week	71 (20.7)	20 (28.2)		
	>Once a week	88 (25.7)	36 (40.9)		
Raw fruit and vegetable eating	Never	85 (24.9)	29 (34.1)	7.06	0.029*
	At least once a day	56 (16.4)	24 (42.9)		
	At least once a week	201 (58.8)	51 (25.4)		
Drinking water source	Tap water	282 (82.5)	86 (30.5)	0.01	0.939
	Non-tap water	60 (17.5)	18 (30.0)		
Hand washing before eating	Yes	322 (94.2)	97 (30.1)	0.21	0.646
	No	20 (5.8)	7 (35.0)		
Hand washing after toilet	Yes	322 (94.2)	94 (29.2)	3.85	0.050*
	No	20 (5.8)	10 (50.0)		
Cigarette smoking	Yes	18 (5.3)	4 (22.2)	0.60	0.438
	Never	324 (94.7)	100 (30.9)		
Tea consumption	Yes	232 (67.8)	65 (28.0)	1.95	0.163
	No	110 (32.2)	39 (35.5)		
Coffee consumption	Yes	211 (61.7)	70 (33.2)	1.99	0.158
	No	131 (38.3)	34 (26.0)		
ABO Blood group	Type A	87 (25.4)	24 (27.6)	10.35	0.016*
	Type B	96 (28.1)	25 (26.0)		
	Type O	137 (40.1)	53 (38.7)		
	Type AB	22 (6.4)	2 (9.1)		
Rhesus (Rh)	Rh + ve	326 (95.3)	96 (29.4)	3.04	0.081
	Rh-ve	16 (4.7)	8 (50.0)		

Key: * = Significant associations exist

### Prevalence of *H. pylori* infection

HpSA and Anti-*H.pylori* IgG detection rate were 30.4 and 60.5%, respectively. Eighty-eight (25.7%) and 119 (34.8%) participants were positive and negative for both tests, respectively. Kappa measures of agreement between results of HpSA testing and IgG antibody serology was 0.271 (*p* < 0.0001, 95% CI: 0.191–0.351), but it was not strongly convincing since discordant results were observed for 135 individuals (39.5%): 16 (4.7%) were positive on HpSA test but negative by serology and 119 (34.8%) were IgG seropositive but had negative HpSA tests.

### Socio-demographic characteristics and feco-prevalence of *H. pylori* infection

Pearson’s chi-square test showed that HpSA detection was significantly associated with number of persons sharing the same bedroom in the family (χ2 = 10.14, *p* = 0.006) and family size in the household (χ2 = 8.25, *p* = 0.004). However, other socio-demographic variables did not show any association (*P* > 0.05) (Table 2).

### HpSA detection with respect to genetic, hygiene condition and lifestyle factors

Pearson’s chi-square test revealed that hand washing after visiting toilet (χ 2 = 3.85, *P* = 0.05), ABO blood phenotype (χ2 = 10.35, *p* = 0.016), alcohol drinking (χ2 = 6.26, *p* = 0.04) and raw fruits and/or vegetables (χ2 = 7.06, *p* = 0.029) eating showed statistically significant association with HpSA detection.

Clearing the possible confounders, multiple logistic regression analysis showed that HpSA detection was significantly associated with ABO blood group type O [AOR = 8.93, 95% CI: 1.79–44.48, *p* = 0.008] and type A [AOR =5.53, 95% CI: 1.08–28.36, *p* = 0.040]; overcrowding: family size greater than three [AOR = 1.83, 95% CI: 1.10–3.05, *p* = 0.02] and greater than three persons sharing the same bed room in the family [AOR = 2.91, 95% CI: 1.39–6.11, *p* = 0.005]. A multivariate analysis also clearly noted that alcohol consumption greater than once a week can increase the risk of *H. pylori* infection by 2.7 times as compared to those who had never drank alcohol [AOR = 2.70, 95% CI: 1.49–4.89, *p* = 0.001]. However, other variables as shown in Table 3 didn’t show any association with antigen detection.

**Table 3:** Regression analysis of factors associated with feco-prevalence of *H. pylori* among symptomatic patients in Dessie Referral Hospital, Northeast Ethiopia, 2016 (*n* = 342)

Variable	Freq	HpSA (+) = no/%	COR(95%CI)	AOR(95%CI)	*p*-value
Family size					
< 4	175	41(23.4)	ref	ref	
≥ 4	167	63(37.7)	1.98(1.24--3.17)	1.83(1.10--3.05)	0.020*
Hand washing after toilet					
Yes	322	94(29.2)	ref	ref	–
No	20	10(50.0)	2.43(0.98--6.02)	2.5(0.92–6.79)	0.073
Eating raw fruit and vegetable					
Never	85	29(34.1)	ref	ref	
At least once a day	56	24(42.9)	1.45(0.72--2.90)	1.38(0.64--2.96)	0.407
At least once a week	201	51(25.4)	0.66(0.38--1.14)	0.64(0.35--1.18)	0.151
Tea consumption					
Yes	232	65(28.0)	ref	–	–
No	110	39(35.5)	1.41(0.87–2.29)	–	–
Coffee consumption					
Yes	211	70(33.2)	ref	–	–
No	131	34(26.0)	0.71(0.44–1.145)	–	–
Alcohol drinking					
Never	183	48(26.2)	ref	ref	
Once/week	71	20(28.2)	1.10(0.60--2.04)	1.35(0.69–2.63)	0.382
> once/week	88	36(40.9)	1.95(1.14--3.33)	2.70(1.49–4.89)	0.001*
ABO Blood group					
Type A	87	24(27.6)	3.81(0.83–17.55)	5.53(1.08–28.36)	0.040*
Type B	96	25(26.0)	3.52(0.77–16.15)	5.01(0.98–25.72)	0.054
Type O	137	53(38.7)	6.31(1.42–28.1)	8.93(1.79–44.48)	0. 008*
Type AB	22	2(9.1)	ref	ref	
Persons per room					
1	77	18(23.4)	ref	ref	
2 to 3	181	49(27.1)	1.22(0.65–2.27)	1.26(0.65–2.43)	0. 495
> 3	84	37(44.0)	2.58(1.31--5.10)	2.91(1.39–6.11)	0.005*

Key: * = Significant association Exist; *AOR* adjusted odds ratio, *COR* crude odds ratio

## Discussion

*H. pylori* infection is an important public health issue globally with high prevalence among developing countries [1–3]. In this study the overall stool antigen and serum IgG detection rate of *H. pylori* infection was 30.4 and 60.5% respectively. Antigen detection rate is comparable with the work of Alim et al. 29.6% [24] and Balan et al. 28.4% [25]. However, a lower prevalence rate was reported from Pakistan 25% [26] and a higher prevalence rate was documented in Turkey 36.6% [27], Japan 56.4% [28], Iran (53.5%) [29] and Ethiopia 50.7–58.3% [19–22]. The observed variation of infection rate might be attributed to differences in study area, sample size, personal hygienic condition, and the socio-economic status as well as life style of persons contributing for risk of infection. Moreover, the difference in the sensitivity and specificity of employed stool antigen tests may also affect the detection rate of infection.

In our study, even though the agreement of *H. pylori* stool antigen and serum IgG detection is statistically significant, it is not strongly convincing [Cohen’s Kappa =0.271, *p* < 0.0001, 95% CI: 0.191–0.351]. Serum IgG detection over estimates *H. pylori* infection (60.5%) as compared to stool antigen detection which is similar to report of Alim et al. 69% [24], Shimoyama et al. 61.4% [28] and Balan et al. 35.8% [25]. The observed high seroprevalence of *H. pylori* infection in our study could be explained due to the following key facts: 1) It is clearly known that during *H. pylori* infection IgM is the first and early antibody to be produced [30], and later IgG develops [31], which remain with high titer and detectable indefinitely even after eradication of *H. pylori*. 2) Detection of IgG to *H. pylori* may not differentiate between current and past infection which leads to an overestimation of the prevalence [30]. Even though *H. pylori* stool antigen detection do not distinguish between live and dead bacteria, it is advantageous to detect early and specific exposure to the bacterium as compared to serology [32].

*H. pylori* feco-prevalence was not significantly associated with age, sex, residence, occupation and educational level (*p* > 0.05). Conflicting reports were documented between association of above mentioned factors and *H. pylori* infection. The significant role of age for acquiring *H. pylori* infection was documented elsewhere [19], but it contrasts our result where no significant association was observed between age and *H. pylori* infection. Similarly, previous studies conducted in Ethiopia showed no significant association between age and *H. pylori* infection [22, 33]. In the current study, there is no significant association between gender and *H. pylori* infection, which is in agreement with other previous studies that indicated no gender difference in *H. pylori* infection status [19, 33]. However, some authors indicated that female [22] and male [21] were at higher risk of infection against their counter parts.

In our study, residence and marital status were not significantly associated with feco-prevalence. Previous reports in Ethiopia also showed no significant association with marital status [21, 22] and residence [21]. However, higher prevalence rate of *H. pylori* infection was observed among individuals living in rural and suburban area [22, 34], which differs from our result. This might be due to high number of (61.4%) urban dwellers in our study. In our study, stool antigen detection also did not show any significant association with type of occupation which is in line with previous study in Ethiopia [22], but it contradicts to other study where agrarians were more affected [21]. This discrepancy could be due to the geographical as well as socio-economic differences, which may lead to occupational difference. Moreover, majority of our study participants were urban dwellers that are not involved in agricultural practice. Likewise, no statistically significant association was observed between *H. pylori* feco-prevalence and educational attainment, which is in agreement to other studies in Ethiopia [21, 22].

*H. pylori* feco-prevalence was significantly associated with large (> 3) family size and number of persons (> 3) sharing the same bed room in the household. This is similar to other findings where large family size significantly associated with *H.pylori* infection [21, 34]. However, it differs from other study where no significant difference was detected with respect to family size [22]. This variation may be due to sample size difference. Moreover, factors contributing for the transmission of a bacterium may differ with geographical location and study population. Scholars also indicated that crowding condition is a risk for *H. pylori* infection [35] which could explain the observed significant association in this study.

This study also showed a significant association between ABO blood groups and stool antigen detection. Individuals with blood group type O were about nine times (Table 3) at higher risk of infection as compared to blood group type AB, which is contrary to other studies that did not show any significant association [21, 22, 33, 34]. However, a stratified meta-analysis indicated that O blood group of dyspeptic patients were significantly associated with *H. pylori* infection [36]. The association of blood group type O and *H. pylori* infection could be related to the expression of carbohydrate receptors of *H. pylori* into gastric tissues in greater quantities among individuals with O blood group [37].

There are conflicting reports on the association between alcohol consumption and *H. pylori* infection. In Ethiopia, Kibru et al. [19] reported that alcohol consumption protects against *H. pylori* infection but Dilnessa et al. [22] showed no significant association in his report. Furthermore, frequent consumption of alcohol (>once/week) showed no significant association but less frequent consumption (<once/week) was protective [21]. In Germany, an inverse relationship was also documented between amount of alcohol consumption and *H. pylori* infection [38, 39] as moderate amount of alcohol consumption in the form of wine and beer protects against *H. pylori* infection [40]. However, the current study is in opposition to previously mentioned studies [19, 21, 22, 38–40] as frequent consumption of alcohol (>once/week) was significantly associated with feco-prevalence of *H. pylori* (*p* = 0.001). But our result is in line with previous report as large amount of alcohol consumption is positively associated with active *H. pylori* infection [41].

The reason for these contradictory results might be due to difference in types and amounts of consumed alcoholic beverages. In Ethiopia, common local alcoholic drinks are “Tella”, “Teji” “Araki” and “beer” which are quite different in alcohol content. The significant association in our study could be explained due to the hypothesis that heavy alcohol consumption facilitates *H. pylori* infection by damaging the gastric mucosa. Besides the damaged gastric mucosa, bacterial adherence and host factors may also be involved in the synergistic effect for infection.

*H. pylori* stool antigen detection was not statistically associated with coffee consumption which is similar to previous reports from Ethiopia [22] and England [40], but differs from Germany where coffee consumption showed a positive dose response relation with active *H. pylori* infection [38]. This contradictory result might be due to variation in type, frequency as well as amount of daily consumed coffee which needs further study. In our study, smoking also did not show any significantly association with *H. pylori* feco-prevalence which is similar to other studies in Ethiopia and elsewhere [21, 22, 38]. This is possibly attributed to *H. pylori* eradication by increasing gastric acid secretion by smoking. Similarly, no significant association was observed between source of drinking water and *H. pylori* infection which is in line with previous studies in Ethiopia [21, 22]. In the current study, the observed lower contribution of source of water to *H. pylori* infection is probably due to type of study participants in which majority of them used pipe water for their daily consumption.

The current study showed no significant association between *H. pylori* infection and raw fruits and vegetables consumption which is in line with previous study [21]. No statistically significant association was also observed between tea consumption and *H. pylori* infection. Lack of association of above factors might be due to difference in types and amount of consumed tea, fruits and vegetables that may have different spectrum of antibacterial activity. Further analytical study designed to determine the effect of quantity and ingredient analysis is, therefore, warranted to document the real association of *H. pylori* infection, fruits, vegetables and tea consumption.

There are a few limitations of this study. The study population was only symptomatic patients presented to the hospital, which limits the actual prevalence of infections and do not totally reflect the number of infected individuals in the community. Besides cross-sectional, all information on life style factors was collected by a self-administered questionnaire without determination of amount, types and biological markers. As compared to currently recommended stool antigen test (ELISA), relatively less sensitivity and specificity of the currently utilized test could underestimate and overestimate the burden of the disease respectively. Moreover, the small sample size may have an impact on the observed association variables, and thus, interpretations should be made cautiously.

## Conclusion

The overall *H. pylori* stool antigen and anti-*H. pylori* IgG detection rate were 30.4 and 60.5% respectively. Even though statistically significant, the agreement between results of HpSA testing and IgG antibody serology was not strongly convincing. Feco-prevalence was significantly associated with overcrowding, alcohol consumption, and ABO blood group. However, no significant association observed between other sociodemographic variables, lifestyle factors and *H.pylori* stool antigen detection. Further studies with representative larger numbers of subjects should address the impact of life style factors such as smoking, and drinking practices particularly the type and amount of daily consumption of tea, coffee, alcoholic beverage; types of fruits and vegetables taken that might affect incidence of *H. pylori* infection.

## Abbreviations

AOR: Adjusted oddis ratio
COR: Crude oddis ratio
HpSA: *H.pylori* stool antigen
IgG: Immunoglobulin G
MALT: Mucosal-Associated lymphoid tissue
PCR: Polymerase chain reaction
PPI: Proton pump inhibitor
RUT: Rapid urease test
UBT: Urea breath test
